# Dielectric Spectroscopy Shows a Permittivity Contrast between Meningioma Tissue and Brain White and Gray Matter—A Potential Physical Biomarker for Meningioma Discrimination

**DOI:** 10.3390/cancers15164153

**Published:** 2023-08-17

**Authors:** Anton Kordić, Antonio Šarolić

**Affiliations:** 1 Department of Neurosurgery, University Hospital Centre Zagreb, 10000 Zagreb, Croatia; 2 FESB, University of Split, 21000 Split, Croatia; antonio.sarolic@fesb.hr

**Keywords:** dielectric spectroscopy, meningioma, human brain white matter, human brain gray matter, tumor tissue discrimination, dielectric contrast biomarker, complex permittivity measurement, microwave gigahertz (GHz) frequency range, open-ended coaxial probe

## Abstract

**Simple Summary:**

Meningiomas are the most common primary central nervous system tumors, and surgery is their primary treatment of choice. The surgical intervention aims for the maximum extent of tumor resection whilst preserving the brain’s neurological functions. The effectiveness of such intervention thus depends on the capability to intraoperatively discriminate between the meningioma tissue and the surrounding brain white matter and gray matter. This study investigated dielectric contrast as a potential physical biomarker for meningioma discrimination. Dielectric contrast is based on the difference in complex permittivity, a physical property of tissues, measurable by dielectric spectroscopy. This study shows that the dielectric contrast is relevant as a potential physical biomarker to discriminate the meningioma tissue from the surrounding brain tissues. Such a biomarker has a meaningful potential to make a positive impact on the management of neurooncological patients in terms of intraoperative tumor discrimination, as well as future diagnostic or therapeutic applications.

**Abstract:**

The effectiveness of surgical resection of meningioma, the most common primary CNS tumor, depends on the capability to intraoperatively discriminate between the meningioma tissue and the surrounding brain white and gray matter tissues. Aiming to find a potential biomarker based on tissue permittivity, dielectric spectroscopy of meningioma, white matter, and gray matter ex vivo tissues was performed using the open-ended coaxial probe method in the microwave frequency range from 0.5 to 18 GHz. The averages and the 95% confidence intervals of the measured permittivity for each tissue were compared. The results showed the absence of overlap between the 95% confidence intervals for meningioma tissue and for brain white and gray matter, indicating a significant difference in average permittivity (*p* ≤ 0.05) throughout almost the entire measured frequency range, with the most pronounced contrast found between 2 GHz and 5 GHz. The discovered contrast is relevant as a potential physical biomarker to discriminate meningioma tissue from the surrounding brain tissues by means of permittivity measurement, e.g., for intraoperative meningioma margin assessment. The permittivity models for each tissue, developed in this study as its byproducts, will allow more accurate electromagnetic modeling of brain tumor and healthy tissues, facilitating the development of new microwave-based medical devices and tools.

## 1. Introduction

Meningiomas are tumors that are derived from the meningothelial cap cells [1] of the arachnoid mater, and they are the most common primary central nervous system (CNS) tumors, accounting for about 37.6% of all primary CNS tumors [2]. In the United States, the prevalence rate of meningioma is 97.5/100,000 [3], and it is estimated that meningiomas occur in 0.9% to 1.0% of all individuals between 50 and 66 years [4]. According to the World Health Organization (WHO), these tumors are classified into three grades, and the majority of meningiomas are grade 1 [1]. The clinical presentation of meningiomas, like in other CNS tumors, depends primarily on the size and the location of the meningioma. Therefore, a wide spectrum of clinical manifestations, like headaches, epileptic seizures, focal neurological deficits, personality changes, and an altered level of consciousness, can occur [3]. For the majority of patients with symptomatic and accelerated growing meningiomas, surgery is the primary treatment of choice [5]. The gold standard for meningioma surgery is the maximum extent of resection with minimum morbidity whilst preserving neurological function. Surgery can provide a 5-year progression-free survival rate of 90%, but it is impacted greatly by the achieved extent of tumor resection [6,7,8]. However, complete tumor resection, the main prerequisite for low tumor recurrence rate, may be hindered by a number of factors including, e.g., tumor location, involvement of nearby neurovascular structures, and brain invasion [1]. Surgical treatment may be even more demanding in cases of tumor recurrence and post-irradiation changes when the identification of the tumor may present a significant challenge [9]. Specifically, it is often difficult to determine the tumor border, i.e., to distinguish the tumor from the tissue surrounding the tumor [10]. Consequently, the completeness of surgical resection depends on the ability of a surgeon to discriminate between the meningioma tissue and the surrounding brain tissues, namely, brain white matter and gray matter. Various techniques exist for intraoperative detection and discrimination of meningioma, some of them are described in the following: (a) intraoperative neuropathological frozen section diagnostics, which is time-delayed and invasive (requiring tissue excision), and agreement with final histopathology diagnoses may actually vary [11]; (b) intraoperative MRI or CT scan, which are time-consuming, not available in the majority of neurosurgical centers, and associated with high costs [12]; (c) fluorescence-guided (5-ALA) surgery, which has been shown to assist tumor identification and improve tumor resection for meningiomas, but for now its clinical usefulness is still questionable [13,14,15]. A few spectroscopic techniques are under investigation for meningioma tissue diagnostics and tumor discrimination, such as nuclear magnetic resonance [16], infrared spectroscopy [17], and Raman spectroscopy [18]. Among the previously mentioned methods, Raman spectroscopy is especially interesting as it has multiple applications in biomedicine and has already been proposed for use in neurosurgery since 1990 [19]. It gives quantitative information regarding the molecular composition of biomaterial/tissue by means of vibrational spectroscopy and it includes: spontaneous Raman spectroscopy, coherent anti-Stokes Raman scattering microscopy, and stimulated Raman histology [20]. Raman spectroscopy methods were used in studies regarding in vivo intraoperative brain cancer detection [21], ex vivo differentiation between glioblastoma and a healthy brain [22], intraoperative histopathology analysis of surgical specimens [23,24], and stereotactic brain tumor biopsies [25]. Regarding meningioma studies specifically, Raman imaging was used for meningioma tumor grading [26] and intraoperative meningioma and dura matter discrimination [27].

The question arises whether discrimination between the tumor and the surrounding tissues can be performed based on the tissues’ dielectric permittivity as an inherent and measurable physical property. Biological tissues, like other materials, can be dielectrically characterized to describe their interactions with the applied electromagnetic field. Dielectric permittivity of a material is a complex physical quantity that quantifies two phenomena occurring when a material is exposed to an electromagnetic field: (1) the material’s ability to store electric energy by internal polarization, and (2) the associated energy loss in the material. The former is described by the real part of the complex permittivity and the latter by the imaginary part of the complex permittivity. Permittivity is temperature- and frequency-dependent. In the microwave frequency range, tissue permittivity is dominantly determined by the chemical composition of tissue, i.e., different types of ions and molecules in the intracellular and extracellular space. As a result of the delay between the applied electromagnetic field and the tissue polarization, there are three characteristic dielectric dispersion types: α-, β-, and γ-dispersions [28,29], which occur from low frequencies to a few hundred gigahertz range. α-dispersion occurs at frequencies lower than 10 kHz and is related to the migration of cell ions in the tissue and surface admittance. β-dispersion occurs around the megahertz region and is related to polarization at the cell membrane interface in the tissue. γ-dispersion occurs in the gigahertz (GHz) region and is linked to the rotation of the dipole moment of polarized molecules, especially the water molecules that are the most abundant molecules in the human body. Thus, water is a crucially important contributor to the dielectric properties of tissues in the GHz frequency range, also called the microwave frequency range.

The efficacy of electromagnetic-based medical applications to discriminate between healthy and pathologically changed tissues should be based on dielectric contrast, which should be determined prior to designing such a system. This contrast depends on the type of malignant tissue, the type of healthy tissue, and the frequency applied. Determining the dielectric contrast between a specific tumor tissue and the relevant surrounding tissue requires a dedicated study, analyzing the complex permittivity of both types of tissues. Permittivity analysis is based on dielectric spectroscopy, which is the process of measuring the tissue dielectric permittivity as a complex physical quantity. Such studies on dielectric contrast have been undertaken for only a few tumors so far, namely: breast tumors in the range of 20 kHz–100 MHz [30] and 0.5–20 GHz [31], colon tumors in the range of 2–8 GHz [32] and 0.5–18 GHz [33], prostate tumors in the range of 0.1–100 kHz [34] and 50–270 MHz [35], hepatic tumors in the range of 10 Hz–1 MHz [36] and 0.5–20 GHz [37], metastatic lymph nodes in the range of 1 MHz–4 GHz [38], glioma tumors in the range of 5–500 MHz [39], skin tumors in the range of 0.5–50 GHz [40], oral tumors–squamous cell carcinoma in the range of 10 MHz–20 GHz [41], and thyroid tumors in the range of 200 MHz–10 GHz [42].

Accordingly, regarding the CNS tumors, to the best of our knowledge, glioma is the only intracranial tumor studied for dielectric contrast [39], and the study only refers to frequencies below the microwave range. On the other hand, the microwave frequency range is of primary interest because it is the working range of slim form probes, whose narrow diameter allows probing the tissue with a space resolution required to discriminate between tissues [43]. Actually, in the microwave frequency range, the data on the brain tissue permittivity is dated and scarce, especially when it comes to human tissues [44], while the data on the permittivity of tumors in human CNS is practically non-existent.

The objective of this study was to determine the dielectric contrast between the meningioma tissue and the tissues of brain white and gray matter, by means of dielectric spectroscopy performed on human tissues, in the microwave frequency range. We hypothesize that such contrast exists and that it is relevant as a potential biomarker. Such a biomarker for tissue discrimination could be potentially used for intraoperative meningioma margin assessment and possibly also for other types of diagnostic and therapeutic applications. Furthermore, the permittivity data for each tissue measured in this study, both meningioma tissue and human brain tissues, will be valuable by itself as an advance in knowledge on tissue permittivity. Such advances will enable more realistic electromagnetic modeling of CNS tumors and healthy tissues, which is useful for various biomedical applications of electromagnetic fields in the microwave frequency range.

## 2. Materials and Methods

### 2.1. Complex Permittivity

The dielectric properties of a material describe its physical response to the applied electric field, whose response consists of two physical phenomena: electrical energy storage in the material due to its electric polarization and electrical energy dissipation (losses) in the material due to the conversion of electrical energy to heat. Both phenomena are frequency-dependent. These two phenomena are characterized by the real and the imaginary part (respectively) of the complex physical quantity called dielectric permittivity, or simply, permittivity, denoted by $\hat{\varepsilon }$, expressed in farads per meter [F/m]. The permittivity of a material is commonly normalized to the permittivity of vacuum (*ε*_0_), resulting in a dimensionless complex quantity ${\hat{\varepsilon }}_{r}$ called relative permittivity, defined by its real and imaginary part:

(1) $${\hat{\varepsilon }}_{r}=\frac{\hat{\varepsilon }}{{}_{0}}=\varepsilon_{r}^{\prime }-j\varepsilon_{r}^{\prime \prime }.$$

Throughout this paper, as a common practice, the term “permittivity” is used as an equivalent to the relative permittivity ${\hat{\varepsilon }}_{r}$. Accordingly, the measured permittivity data in this paper is expressed in terms of the real and the imaginary part of the relative permittivity $\varepsilon_{r}^{\prime }$ and $\varepsilon_{r}^{\prime \prime }$, respectively. It is worth noting that some references describe the electrical losses in terms of conductivity *σ* in Siemens per meter [S/m] instead of the dimensionless imaginary part of permittivity $\varepsilon_{r}^{\prime \prime }$. The two quantities are interrelated as follows:

(2) $$\sigma =\omega {}_{0}\varepsilon_{r}^{\prime \prime },$$

where ω stands for the angular frequency.

Consequently, in this paper, the measured permittivity data will be expressed as the real and the imaginary part of the complex relative permittivity, i.e., dimensionless quantities $\varepsilon_{r}^{\prime }$ and $\varepsilon_{r}^{\prime \prime }$, respectively, as a function of frequency.

### 2.2. Cole–Cole Model of Complex Permittivity

Frequency dependence of permittivity is a result of different polarization mechanisms occurring in a material, arising due to the presence of various polarized structures having different relaxation times. Assuming the existence of multiple relaxation regions, the frequency dependence of a biological tissue permittivity is commonly modeled using the Cole–Cole model [45], i.e., the empirical formula that models the dispersion and absorption in lossy dielectrics:

(3) $${\hat{\varepsilon }}_{r}=\varepsilon_{\infty }+\sum_{i=1}^{N}\frac{\varepsilon_{s,i}-\varepsilon_{\infty }}{1+{\left(j\omega \tau_{i}\right)}^{1-\alpha_{i}}}+\frac{\sigma }{j\omega \varepsilon_{0}},$$

where $\varepsilon_{\infty }$ and $\varepsilon_{s,i}$ are the high- and low-frequency asymptotes of the dielectric constant, respectively, for the modeled frequency range and *σ* is the conductivity asymptote at the lower end of the modeled frequency range. *N* is the number of Cole–Cole poles modeling different relaxation regions present in the modeled frequency range, while *τ_i_* is the associated relaxation time and *α_i_* is a measure of the broadening of the *i*-th dispersion. Each relaxation region is the manifestation of a polarization mechanism characterized by a different time constant.

As the dominant relaxation mechanism in the microwave frequency range is the relaxation of the water molecules, in this study, the Cole–Cole model can be reduced to a single-pole model:

(4) $${\hat{\varepsilon }}_{r}=\varepsilon_{\infty }+\frac{\varepsilon_{s}-\varepsilon_{\infty }}{1+{\left(j\omega \tau_{}\right)}^{1-\alpha_{}}}+\frac{\sigma_{}}{j\omega \varepsilon_{0}}.$$

where the parameters of the pole refer to the water relaxation. Accordingly, as one of the outputs of this study, we derived the single-pole Cole–Cole models to fit the averaged measurement data, for each species and each tissue, as an additional representation of our measurement results. The Cole–Cole models were derived to fit the data measured from 0.5 GHz to 18 GHz with the target average error < 5% and max error < 10%, both for $\varepsilon_{r}^{\prime }$ and $\varepsilon_{r}^{\prime \prime }$. Accordingly, the model parameters are valid only in this frequency range and do not necessarily reflect the parameters of the material outside this frequency range.

Although the developed Cole–Cole models for the measured tissues do not have a direct role in our hypothesis testing, they are considered additional valuable byproducts of this study, as they can serve other researchers to model the dielectric parameters of the analyzed tissues in the microwave frequency range when necessary for the development of microwave-based medical applications.

### 2.3. Dielectric Spectroscopy

**Figure 1:**
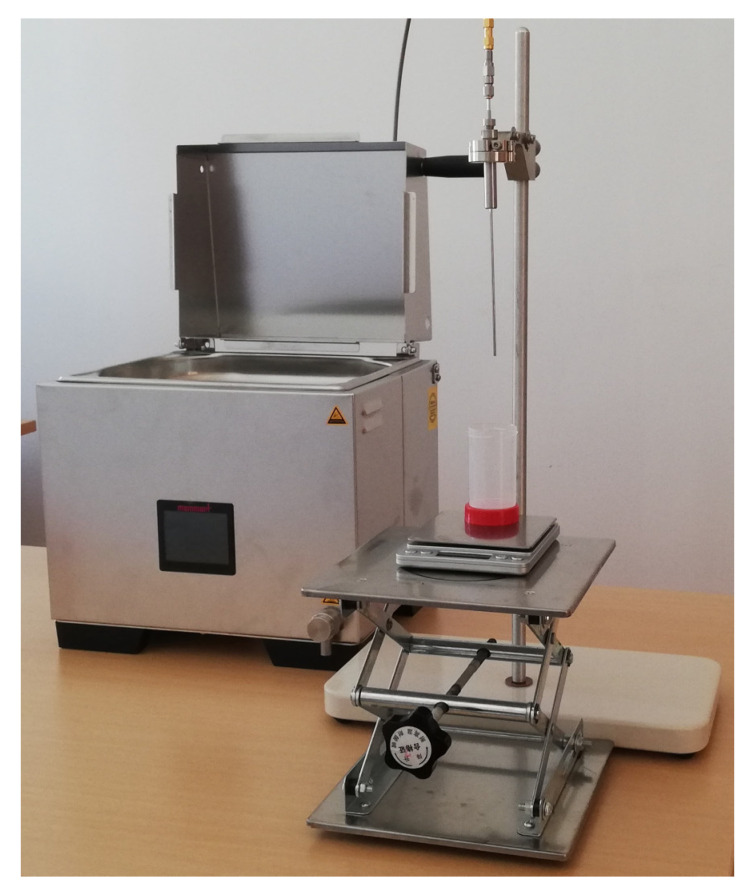
Slim Form Probe on a stand above a sample holder with a fine-tuned contact control.

The dielectric spectroscopy, i.e., the complex permittivity measurement, was performed using the standardized open-ended coaxial probe method, which is described in detail in our previous studies [44,46,47]. It is a non-invasive method (at the sample level), where an open-ended coaxial probe is put in contact with the sample acting as the material under test (MUT) (Figure 1). A Slim Form Probe [46,48] was connected to N9927A FieldFox vector network analyzer (VNA) [49] and controlled by manufacturer-provided software Keysight Materials Measurement Suite; all equipment was manufactured by Keysight Technologies Inc., Santa Rosa, CA, USA. The name Slim Form Probe indicates that its diameter of only 2.2 mm is smaller than most other commercial probes. Accordingly, it is a common choice for biological tissue measurements [43,44] as it allows to differentiate between the tissues in a heterogeneous sample, e.g., to separately measure the white matter or the gray matter on a brain slice.

The frequency range of dielectric spectroscopy performed in this study was from 500 MHz up to 18 GHz, as determined by the lower frequency limit of Slim Form Probe (500 MHz) and the upper-frequency limit of N9927A FieldFox VNA (18 GHz). The measurements were performed with 10 MHz steps, resulting in 1751 frequency points across the whole frequency range.

The measurement setup was calibrated using three known loads: open (probe in the air), short (probe in firm contact with a conductive sheet, i.e., electrically “shorted”), and a known liquid (probe in deionized water). Calibration was performed before starting the measurements on each sample.

Upon delivery of the samples from surgeries and autopsies, they were brought to the temperature of 25 °C by keeping them in the water bath (WTB11, Memmert GmbH, Schwabach, Germany) in sealed containers until the time of measurement. The room temperature was also kept at 25 °C.

The probe was cleaned with 70% ethyl alcohol before starting the measurements on each point on a sample to prevent contaminating the measurement point with any residues from the previous measurements.

In this study, at each point, 3 consecutive measurements were performed, and then the average of those 3 measurements was taken as a measurement result. This procedure removes stochastic noise and obtains more accurate measurements.

### 2.4. Tissues under Test

This study was performed under the ethical approval issued by the Ethics Committee of the University Hospital Centre Zagreb (Class: 8.1-20/48-2, No. 92/21 AG).

**Figure 2:**
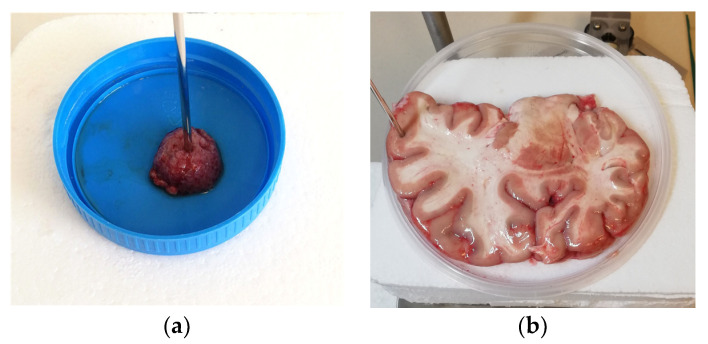
Dielectric spectroscopy. Slim form probe in contact with (**a**) meningioma tissue; (**b**) cortex gray matter tissue on a brain slice.

Meningioma tissues from 5 different tumors were obtained after neurosurgical excisions at the Department of Neurosurgery and measured before putting them in formalin and sending them to pathohistological analysis at the Department of Pathology and Cytology, University Hospital Centre Zagreb. The dielectric spectroscopy procedure was non-destructive for the sample (Figure 2), and it did not interfere with subsequent pathological analysis of the sample. The samples were delivered in closed containers, and no dehydration or decomposition of tissues was noticeable prior to measurement. The excised tumors varied in size, and not all of them were delivered in one piece. Nevertheless, all the measured pieces had dimensions considerably larger than the probe diameter, ranging from ca. 5 mm to ca. 20 mm. Each tumor was measured at as many different points as possible, depending on the size of the excised material, thus the measurements times varied from 0.5 to 2 h after the excision.

White and gray matter human tissues were obtained from five hospital autopsies at the Department of Pathology and Cytology, University Hospital Centre Zagreb. Three brains were measured specifically for this study, labeled hereafter as H3, H4, and H5. Two brains, H1 and H2, were measured already for our previous study [44]. However, brain H2 was not used for this study due to dispersion of its results for the white matter being considerably larger than for the other four brains, which were found to have similar standard deviations of their permittivity. We were not able to reconstruct the reasons for this deviation, whether it was due to inherent properties of the tissue or due to measurement process and tissue handling imperfections for that specific brain, thus we decided not to use this data in this study.

The autopsies were performed within 1–2 days from the time of death. The dielectric spectroscopy was performed within a period of a few hours after the autopsy. Prior to autopsy, the bodies were kept in the refrigerator at 4 °C, and the brains were intact in the skull. Neither of the patients had any disclosed brain pathologies noted in their medical record. The human brain samples were delivered in the form of brain slices of around 1.5 cm thickness, containing both white and gray matter. The white and the gray matter were visually distinguishable; thus, the dielectric spectroscopy was performed on visually homogeneous regions of white and gray matter on each brain slice (Figure 2). White and gray matter from each brain slice were measured at as many different points as possible.

The final number of analyzed measurement points per tissue was 38 for meningioma, 42 for white matter, and 51 for gray matter, and these numbers were regarded as the sample sizes for the statistical analysis of each tissue type.

### 2.5. Statistical Analysis

For each type of tissue (meningioma, white matter, or gray matter), dielectric spectroscopy results of the total number of measurement points per tissue (i.e., the statistical sample for each tissue type) were tested for normality using the Jarque–Bera test (*p* ≤ 0.05), and they were tested separately for the real and the imaginary part of complex permittivity ($\varepsilon_{r}^{\prime }$ and $\varepsilon_{r}^{\prime \prime }$, respectively). After confirming that the results show normality across all measurement points, the mean value and the 95% confidence interval of both $\varepsilon_{r}^{\prime }$ and $\varepsilon_{r}^{\prime \prime }$ were calculated for each tissue type and presented graphically as a function of frequency. The 95% CI for each tissue type was calculated using *t*-distribution for the respective sample size for each tissue type: 38, 42, and 51 observations for meningioma, white matter, and gray matter, respectively.

The null hypothesis H0 of this study is that there is no statistically significant difference between the average permittivity of meningioma tissue and brain white and gray matter tissues, both for the real part and the imaginary part of permittivity, in the microwave frequency range. The alternative hypothesis H1 is that the permittivity of meningioma tissue is statistically significantly different from the permittivity of brain white and gray matter tissues, either in their real part or imaginary part, in the microwave frequency range. A statistically significant difference (supporting the alternative hypothesis H1) would demonstrate a dielectric contrast between the meningioma tissue and brain white and gray matter tissues, which could serve as a novel biomarker for meningioma discrimination from the surrounding tissues. The hypotheses were tested by comparing the 95% confidence intervals of meningioma permittivity against the white and gray tissue permittivity, respectively, separately for $\varepsilon_{r}^{\prime }$ and $\varepsilon_{r}^{\prime \prime }$. The absence of overlap between the 95% confidence intervals indicates a significant difference in average permittivity (*p* ≤ 0.05).

## 3. Results

### 3.1. Brain White and Gray Matter Permittivity

**Figure 3:**
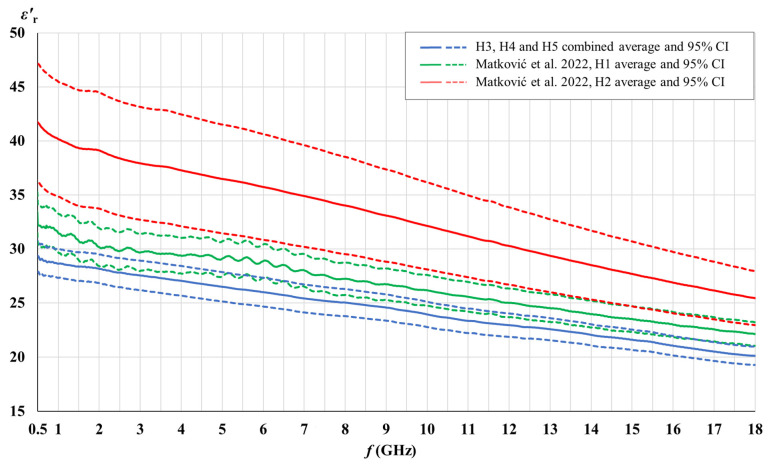
Comparison of white matter permittivity (real part) of the measured brains with literature [44].

**Figure 4:**
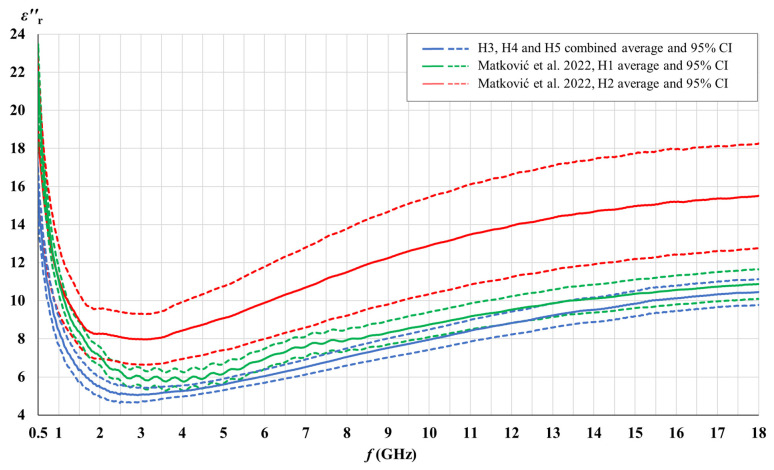
Comparison of white matter permittivity (imaginary part) of the measured brains with literature [44].

**Figure 5:**
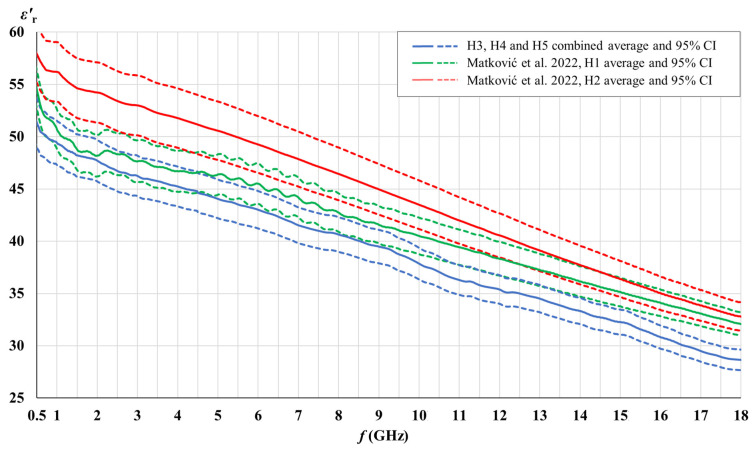
Comparison of gray matter permittivity (real part) of the measured brains with literature [44].

**Figure 6:**
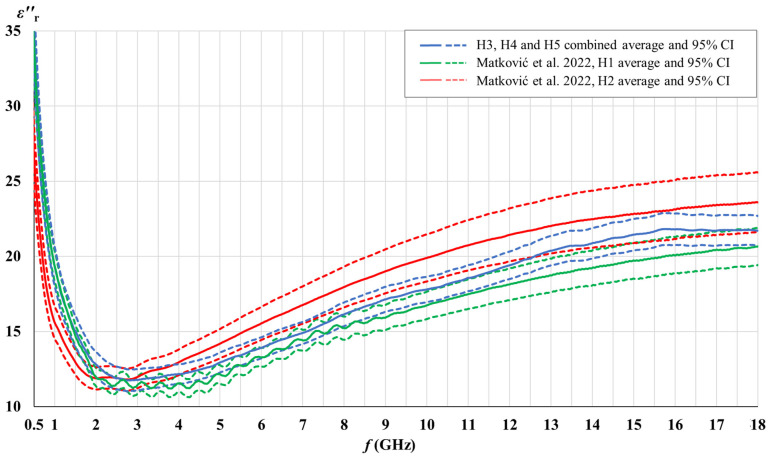
Comparison of gray matter permittivity (imaginary part) of the measured brains with literature [44].

Figure 3, Figure 4, Figure 5 and Figure 6 show the comparison of the measured complex permittivity of white matter and gray matter measurement points over the five measured brains: H1 and H2 already measured in our previous study [44], along with H3, H4, and H5 added for this study. The real and the imaginary part of permittivity are shown with their averages and the corresponding 95% CI.

H3, H4, and H5 measurement points results converged with a narrow confidence interval and are therefore shown combined; in comparison to points on H1 and H2, which are shown separately.

Figure 3, Figure 4, Figure 5 and Figure 6 show that the permittivity of white matter and gray matter measurement points on brains H3, H4, and H5 agrees with the brain H1 as their confidence intervals overlap over the entire frequency range (white matter $\varepsilon_{r}^{\prime }$, gray matter $\varepsilon_{r}^{\prime \prime }$) or at least over the major part of the frequency range (white matter $\varepsilon_{r}^{\prime \prime }$, gray matter $\varepsilon_{r}^{\prime }$). The width of the confidence intervals of H1, H3, H4, and H5 permittivity, i.e., the standard deviations for points on those brains, were also similar. Also, the normality test showed the normal distribution of data for white matter and for gray matter over the entire sample consisting of H1, H3, H4, and H5 points, both for $\varepsilon_{r}^{\prime }$ and $\varepsilon_{r}^{\prime \prime }$. On the other hand, the dispersion of H2 permittivity results for the white matter was considerably larger than for the other three brains. The larger dispersion of H2 was already noticed in [44] with respect to brain H1, and in this study, we additionally confirmed that H2 results show a considerably larger standard deviation than the other four measured brains, especially for white matter measurements. In order to avoid any potential inadvertent inconsistencies, we treated H2 as an outlier brain and did not use H2 data in this study. Accordingly, the final samples for white and gray matter to be analyzed further in this study were composed of the measurement points measured on H1, H3, H4, and H5.

**Figure 7:**
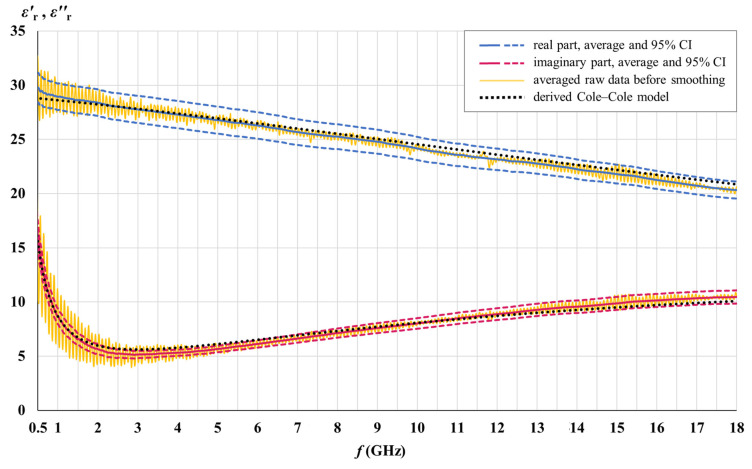
Measured white matter permittivity for the entire sample.

**Figure 8:**
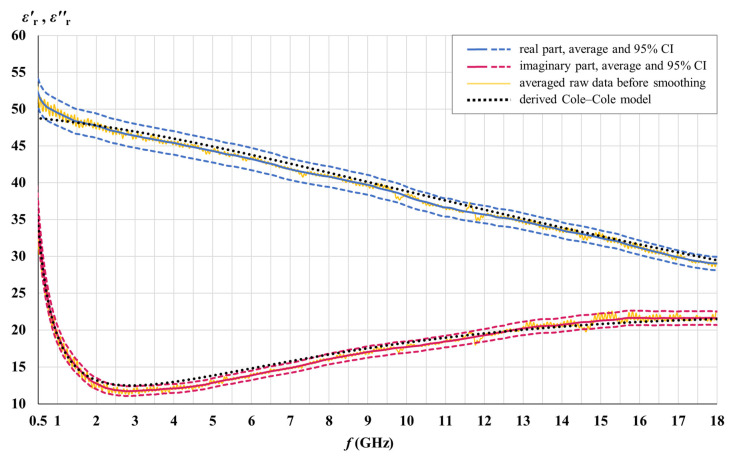
Measured gray matter permittivity for the entire sample.

The real and the imaginary part of the measured white matter and gray matter permittivity are shown in Figure 7 and Figure 8 for the entire samples of each tissue (42 and 51 measurement points for white matter and gray matter, respectively, over the brains H1, H3, H4, and H5). The real and the imaginary part of permittivity are shown with their averages and the corresponding 95% CIs along with the Cole–Cole models derived from the measured results. Averaged raw data shows oscillations arising as a result of measurement imperfections. The actual permittivity is found after smoothing the averaged raw data.

### 3.2. Meningioma Tissue Permittivity

**Figure 9:**
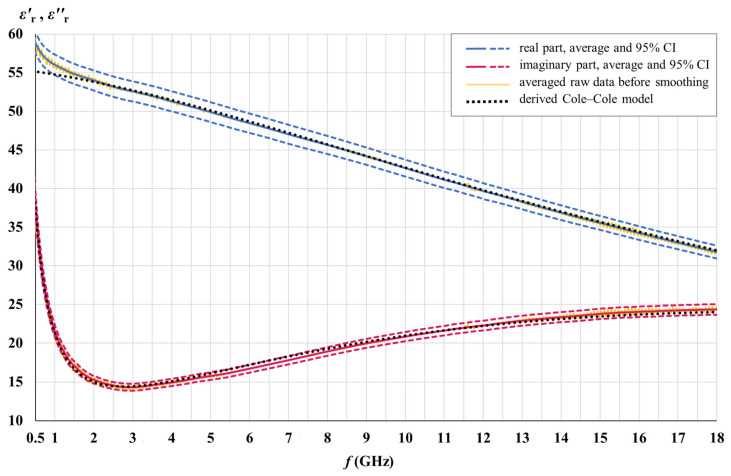
Measured meningioma tissue permittivity for the entire sample.

Figure 9 shows the real and the imaginary part of the measured meningioma tissue permittivity for the entire statistical sample consisting of 38 measurement points.

The real and the imaginary part of permittivity are shown with their averages and the corresponding 95% CIs, along with the Cole–Cole model derived from the measured results.

### 3.3. Dielectric Contrast between the Meningioma Tissue and Brain White and Gray Matter

**Figure 10:**
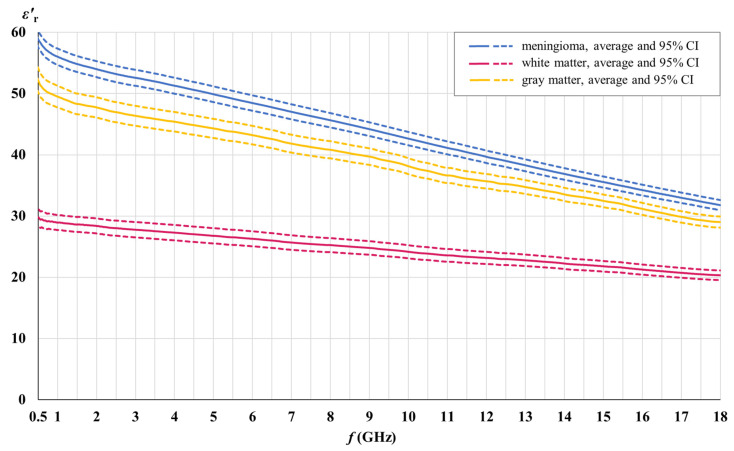
Dielectric contrast between the meningioma tissue and brain white and gray matter; real part of permittivity.

**Figure 11:**
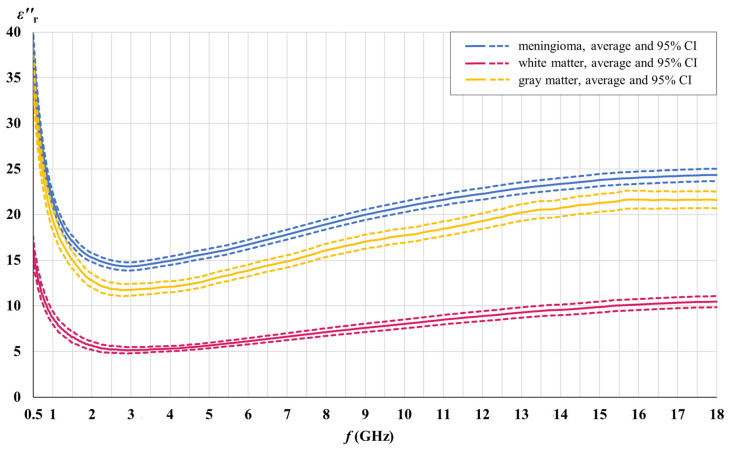
Dielectric contrast between the meningioma tissue and brain white and gray matter; imaginary part of permittivity.

Figure 10 and Figure 11 show the dielectric contrast between the meningioma tissue and brain white and gray matter separately for the real and the imaginary part of the measured permittivity. The real and the imaginary part of permittivity are shown with their averages and the corresponding 95% confidence intervals.

According to Figure 10, there is no overlap between the 95% confidence intervals for the real part of the permittivity for meningioma tissue and for brain white and gray matter. Figure 11 shows a similar absence of overlap for the imaginary part of the permittivity, except a slight overlap between meningioma tissue and gray matter, which exists only in a narrow range of the lowest frequencies. Data analysis shows that the observed overlap exists only at frequencies less than 700 MHz. The presented data supports the alternative hypothesis H1 and allows us to reject the null hypothesis H0.

### 3.4. Cole–Cole Models and Supplementary Materials

Cole–Cole models were developed to fit the measured averages, as described in Section 2.2. The derived parameters for each tissue are given in Table 1 and used with Equation (4) to calculate the Cole–Cole lines in Figure 7, Figure 8 and Figure 9.

The measured permittivity values presented in Figure 7, Figure 8 and Figure 9 are made available as downloadable Supplementary Materials accompanying this paper. The raw data and the data after smoothing are given for each tissue (meningioma, white matter, and gray matter), both for the real and for the imaginary part of permittivity. The measured data is accompanied by the permittivity calculated by (4), using the derived Cole–Cole models for each tissue, with the parameters from Table 1.

## 4. Discussion

### 4.1. Dielectric Contrast

The objective of this study was to determine the existence of a dielectric contrast between the meningioma tissue and the tissues of brain white and gray matter, by means of dielectric spectroscopy performed on human tissues, in the microwave frequency range. We hypothesized that such contrast exists and that it is relevant as a potential biomarker. The results presented in Figure 10 and Figure 11 clearly allow us to reject the null hypothesis H0 and support the alternative hypothesis H1 that the average permittivity of the meningioma tissue is different from the permittivity of brain white and gray matter tissues, either in their real part $\varepsilon_{r}^{\prime }$ or imaginary part $\varepsilon_{r}^{\prime \prime }$, in the microwave frequency range. This difference is statistically significant (*p* ≤ 0.05), as shown by the absence of overlap between the 95% confidence intervals.

The minor overlap between meningioma tissue and the imaginary part of the gray matter of the permittivity in a narrow range of the lowest frequencies < 0.7 GHz is negligible, considering the clear absence of overlap in the rest of the analyzed microwave range up to 18 GHz. The only consequence of this small overlap is that frequencies less than 0.7 GHz cannot be used for the proposed biomarker to compare meningioma and gray matter, for the imaginary part of the permittivity only. The analysis shows that a significant dielectric contrast, usable as a biomarker, exists above 0.7 GHz, while the largest, hence also the most usable contrast, is well above 0.7 GHz.

**Figure 12:**
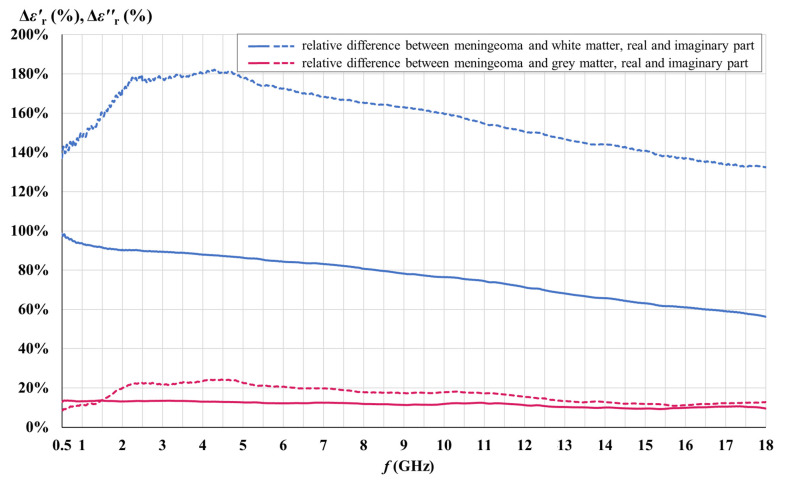
Relative dielectric contrast between the meningioma tissue and brain white and gray matter.

Figure 12 shows the relative dielectric contrast between the meningioma tissue and brain white and gray matter, calculated separately for the real and the imaginary part of the measured permittivity, as a percentage difference:

(5) $${∆\varepsilon }_{r}^{}\left(\%\right)=\frac{{∆\varepsilon }_{r\left(meningioma\right)}^{}-{∆\varepsilon }_{r\left(white/gray\;matter\right)}^{}}{{∆\varepsilon }_{r\left(white/gray\;matter\right)}^{}}.$$

Our results demonstrate that the measured meningioma tissue had on average 76.7% and 157.6% higher values of $\varepsilon_{r}^{\prime }$ and $\varepsilon_{r}^{\prime \prime }$, respectively, when compared to white matter, as well as 11.6% and 16.7% higher values of $\varepsilon_{r}^{\prime }$ and $\varepsilon_{r}^{\prime \prime }$, respectively, when compared to gray matter, for the whole measured frequency range.

For the white matter $\varepsilon_{r}^{\prime }$, the maximum contrast of 98% was found at the lowest measured frequency with the contrast steadily decreasing towards 56% at the highest measured frequency. For $\varepsilon_{r}^{\prime \prime }$, the maximum contrast was found between 2 GHz and 5 GHz, with a maximum of 182%, while the minimum contrast was 132% at the highest measured frequency.

For the gray matter $\varepsilon_{r}^{\prime }$, the maximum contrast of 14% was found at the lowest measured frequency, decreasing slowly and steadily towards 9% at the highest measured frequency. For $\varepsilon_{r}^{\prime \prime }$, the maximum contrast was found between 2 GHz and 5 GHz, with a maximum of 24%. The minimum contrast was 8% at the lowest frequency of 0.5 GHz; however, the contrast between the meningioma tissue and gray matter is not statistically significant anyway for $\varepsilon_{r}^{\prime \prime }$ below 0.7 GHz.

Based on the above observations, the most sensible and usable frequency range for a novel permittivity-based meningioma biomarker to discriminate meningioma from white and gray matter would be between 2 GHz and 5 GHz, where the $\varepsilon_{r}^{\prime }$ contrast for white matter is in the range of 86–90% and stays at 13% for gray matter, while the $\varepsilon_{r}^{\prime \prime }$ contrast for white matter is in the range of 171–182% and is in the range of 20–24% for gray matter.

Compared to the gray matter tissue, a significantly higher average difference in permittivity between the measured tumor tissues and the white matter tissue is evident. It is known that the complex permittivity increases proportionally to the water content of a tissue [50]. Thus, this distinction between white and gray matter permittivity difference in comparison to tumor tissues can be explained by the fact that the white matter water content is around 69% in comparison to the 83% water content of the gray matter [50]. In general, the structure and permeability of a tumor cell membrane are altered with respect to healthy cells, resulting in the accumulation of sodium and water inside the cell [51]. Therefore, higher values of complex permittivity for the measured tumor tissues can be caused by higher water content in tumor tissues, protein hydration, vascularization, and tumor cell density [28,34,36,37,52,53].

### 4.2. Comparison with the Published Literature

Due to various problems in undertaking the dielectric measurement studies of human tumor tissue samples, primarily regarding the demanding logistics of systematic sample collection, handling, and timely measurement within hospital facilities, the literature regarding the dielectric characterization of tumor tissues is scarce [54]. To the best of our knowledge, this is the first study to report meningioma permittivity values. Therefore, our measurement results for meningioma permittivity cannot be directly compared with a corresponding set of previously measured data. Nonetheless, the primary goal of our study was to determine the relative dielectric contrast between the tumor tissue of meningioma and the white and gray matter tissue. Thus, regarding the comparison with the previously published literature, it is reasonable to analyze the relative dielectric contrast measured in this study and compare it to the dielectric contrasts reported in published studies on other types of tumors.

In Table 2 we compared the average dielectric contrast measured in this study with the dielectric contrasts found between various other reported tumor tissues and their respective surrounding tissues in the temperature and frequency range corresponding as closely as possible to our parameters.

In Table 2, one can note that the dielectric contrast between tumor tissues and respected surrounding tissues ranges from 8% to 100% for previously published studies. Our findings are within the range of previously published studies in Table 2, except for significantly higher dielectric contrast in the imaginary part of permittivity between meningioma tissue and white matter tissue, which is on average 157.6%. Table 2 also shows that the previously published studies show the trend of the imaginary part of permittivity having higher dielectric contrast than the real part of permittivity. This observation is also consistent with our findings and suggests that the dielectric contrast in the imaginary part of permittivity may be superior for discriminating between a tumor and the surrounding tissue.

The study by Lu et al. [39] is, to the best of our knowledge, the only published study analyzing dielectric contrast between a tumor and white matter tissue; however, it was conducted in a much lower and narrower frequency range. Lu et al. [39] reported that the average dielectric contrast between glioma and white matter is 30% for the real and imaginary parts of permittivity. Our results for dielectric contrast between meningioma and white matter significantly exceed those of Lu et al. [39], which can be attributed either to a different tumor or to a different frequency range, or both. Nevertheless, this might suggest that the microwave frequency range analyzed in this study is more convenient to develop a biomarker for tumor discrimination than the frequencies below the microwave range.

### 4.3. Cole–Cole Models

Table 1 brings the parameters of the single-pole Cole–Cole models we derived from the measurement data. Figure 7, Figure 8 and Figure 9 show that the developed Cole–Cole models, within the modeled frequency range of 0.5–18 GHz, fit the measured averages well. Small deviations of the Cole–Cole fitting functions with respect to measurement data are inevitable, and our goal was to keep them within 10% of maximum error, while the average error for the whole frequency range was minimized as much as possible and kept within the target of 5%. At the same time, we strived to keep the Cole–Cole fits within the 95% confidence intervals as much as possible, as well as balance the best fit both for $\varepsilon_{r}^{\prime }$ and $\varepsilon_{r}^{\prime \prime }$ at the same time. Table 3 shows the errors of the Cole–Cole models for each tissue.

With a different combination of the Cole–Cole parameters, we could have achieved an almost perfect fit to only one part of the permittivity, either $\varepsilon_{r}^{\prime }$ or $\varepsilon_{r}^{\prime \prime }$, but in that case, the other part of the permittivity would not be optimally fitted. Hence, various combinations of the Cole–Cole parameters are possible to produce rather good fits in the modeled frequency range, and the combinations given in Table 1 are not the only possible Cole–Cole models.

It is also worth noting that the goal of average and maximum error was set and achieved only for the modeled frequency range, while beyond and above that range the deviations of the model could be much higher. It can be concluded that the derived Cole–Cole parameters in Table 1 are valid only for the modeled frequency range, from 0.5 GHz to 18 GHz, and should not be used to model the tissue permittivity (or its components) outside of this frequency range. If the modeled frequency range were much wider, an optimal fit (i.e., the least-error fit) would be achieved by a different set of Cole–Cole parameters, decreasing the precision of such a model within a limited frequency range such as this one, however.

Permittivity modeling within a limited frequency range is a valid approach, especially when modeling a frequency range with only one dominant relaxation mechanism, such as water dispersion in the microwave frequency range. Similarly, in her seminal study [55], Gabriel gave a separate set of Cole–Cole parameters for the water dispersion range due to its importance, which substantially differs from the Cole–Cole parameters given for the wider frequency range in the same study. Since the single-pole model (4) does not take into account the dispersions other than the water dispersion, the conductivity *σ* is in that case, as well as in our study, attributed not only to the static ionic conductivity but also to the dispersions occurring just below the modeled frequency range not modeled by the single-pole model (4). This is also the reason for the deviations of the Cole–Cole models at the beginning of the modeled frequency range, notable in Figure 7, Figure 8 and Figure 9.

The Cole–Cole models developed in this study are a valuable contribution to the researchers looking to model the brain tissues for various applications, while the special benefit and interest is in the first-ever Cole–Cole model of meningioma permittivity, which, to the best of our knowledge, has never been derived before.

### 4.4. Limitations

The future use of the results of this study refers to the potential development of a novel biomarker to discriminate meningioma from the surrounding tissues in vivo. However, this study was performed on ex vivo tissues, which is a common approach to human tissue dielectric measurements [40,56,57]. This could be considered as a potential limitation of this study if the dielectric permittivity of ex vivo tissues significantly differed from the in vivo tissues. However, a common agreement is that differences between ex vivo and in vivo dielectric properties are more important at lower frequencies (<0.1 GHz), whereas little or no significant differences are observed at higher frequencies [58]. In the frequency range of our measurements, the water content and state dominantly determine the dielectric properties. In relatively recent papers [59,60], the authors demonstrated that dielectric properties of tissues measured from 0.5 GHz to more than 40 GHz in vivo show only small changes in dielectric properties when compared to ex vivo tissues, and that difference in dielectric properties between in vivo and ex vivo tissues was comparable to experimental uncertainty of measurement.

On the other hand, in vivo measurements of human white matter, gray matter, and meningioma could only be performed intraoperatively, during open brain surgery. Considering that the wideband measurement method accuracy essentially depends on a stabilized measurement setup, with precise control of the probe contact with the tissue (Section 2.3), it would be extremely technically demanding to establish such setup control during the surgery. Also, wideband measurements are time-consuming, and even if the setup were successfully deployed, the wideband measurements would prolong the open brain surgery by a considerable time, which is hardly acceptable from a surgical point of view. Longer surgery duration increases the probability of complications related to increased risk of general anesthesia, as well as to other risks, such as surgical site infections and other neurosurgical complications. A study [61] showed that the likelihood of complications increased significantly with prolonged operative duration, and for every 30 min of additional operating time, the risk for complications increases by 14%. This would raise ethical issues in justifying such an intraoperative intervention, considering that ex vivo tissues provide quite accurate information on permittivity. Taking these issues into consideration, it is ethically much more acceptable that a wideband study is performed ex vivo. In future developments of a permittivity-based biomarker, the intraoperative in vivo measurements could be considered to validate these findings only in the narrow frequency bands of interest, with a specially developed probe and setup that would provide both the measurement speed and the required control of the probe/tissue contact.

Considering that there are 15 different histological subtypes [1] of meningioma recognized by WHO, which are organized into three grades (generally reflecting the likelihood of recurrence and the rate of growth based on cytological features), and the fact that in our study we analyzed only five meningiomas consisting of five different subtypes (four were grade I and one was grade II), we acknowledge this as a potential limitation of our study. However, we had a rationale to complete this first study with five meningiomas. According to the WHO grades for meningioma, about 80 to 81% are considered typical or grade I, while 17 to 18% of them are atypical or grade II, and 1.7% are anaplastic or grade III meningiomas [62]. Histopathologically, the most often diagnosed grade I meningioma subtypes are as follows: meningothelial (58.3%), fibroblastic (13.4), transitional (10.2%), and psammomatous (3.84%) [63]. Namely, out of the four grade I meningiomas we measured, one was a meningotheliomatous subtype, one was a transitional subtype, one was fibroblastic, and one was a psammomatous type. The fifth meningioma was a grade II meningioma atypicum. Thus, the five measured meningiomas actually encompassed more than 95% of meningioma subtypes diagnosed in the population. Analyzing our results for meningiomas, we realized that their permittivity converges very tightly, regardless of their grade and type, and despite their differences, the resulting 95% CI is quite narrow. This gave us the confidence to publish the results with five meningiomas. Nevertheless, we certainly aim to measure more meningiomas in the follow-up study (or studies), but we do not expect the future results to deviate significantly from the ones presented in this study.

Another point worth mentioning is that the primary aim of this study is reporting permittivity data and testing the hypothesis on the permittivity contrast between the meningioma tissue and brain white matter and gray matter as a potential biomarker. In that respect, we want to emphasize that the potential development of tissue discriminating procedure and device based on this biomarker is beyond the scope of this study and that the real-life discrimination application may be hindered by the fact that meningioma may cause associated brain oedema proportional to the size of the meningioma [64] and present in 67% of meningiomas [65], thus resulting in the increase of the water content of the surrounding gray and white matter tissues and possibly reducing dielectric contrast. Also, the surrounding healthy brain tissue might be glioticaly altered [66,67,68] (non-specific secondary event to brain damage) and might not have the same dielectric properties as the healthy brain tissue investigated in this study. Therefore, the development of the tissue discriminating procedure and device warrants further studies in order to investigate all of the aforementioned study limitations.

A frequency limitation of this study is that all the results, outcomes, and conclusions are valid only for the analyzed frequency range from 0.5 GHz to 18 GHz.

## 5. Conclusions

This study is the first to provide promising results for the feasible use of complex permittivity data to discriminate meningioma tissue from the brain white and gray matter tissue. The results of this study, obtained by means of dielectric spectroscopy, supported our hypothesis that a dielectric contrast exists between meningioma tissue and brain white matter tissue, as well as between meningioma tissue and brain gray matter tissue, in the microwave frequency range. The found contrast is relevant as a potential physical biomarker to discriminate the meningioma tissue from the surrounding brain tissues by means of permittivity measurement. Such a biomarker could potentially be used for intraoperative meningioma margin assessment by means of a medical device to be developed in the future. The significant differences in both the real and the imaginary part of the permittivity, throughout the microwave frequency range, allow various realizations of a permittivity measurement medical device. The development of such a device will be a topic of our future work.

Further research on tissue discrimination based on differences in dielectric permittivity has a meaningful potential to make a positive impact on the management of neurooncological patients in terms of tumor tissue discrimination, as well as for other future diagnostic or therapeutic applications. In that respect, the tissue dielectric permittivity models, developed in this study as their byproducts, will allow more accurate electromagnetic modeling of tumors and healthy tissues, which can facilitate the development of new medical devices and tools.

## Figures and Tables

**Table 1:** Cole–Cole models’ parameters for the measured white matter and gray matter permittivity.

Tissue Type	$\varepsilon_{s}$	$\varepsilon_{\infty }$	*τ*	*α*	*σ*
White matter	29	1.5	4.7 ps	0.15	0.41 S/m
Gray matter	49	1	7 ps	0.085	0.92 S/m
Meningioma	55.5	1	7.4 ps	0.1	1 S/m

**Table 2:** Average difference in permittivity between tumor tissues and their surrounding tissue with the reported temperature and frequency range.

Authors	Tumor Tissue	f (GHz)	T (°C)	$\Delta \varepsilon_{r}^{\prime }$ (%)	$\Delta \varepsilon_{r}^{\prime \prime }$ (%)
Guardiola et al. (2018) [32]	Colon	2–8 5–8	20–22 20–22	30–100	30–100
Fornes-Leal et al. (2016) [33]	Colon	0.5–18	20–25	8.8	10.6 *
Lazebnik et al. (2007) [31]	Breast	0.5–20	18–27	<10 **	<10 **
Gavazzi et al. (2018) [42]	Thyroid	0.2–10	19.1 ± 1.3	10	8–21 ***
Yu et al. (2020) [38]	Metastatic lymph node	0.001–4	24 ± 2.4	22.2–24.6	24–27.2
O’Rourke et al. (2007) [37]	Liver	0.915 2.45		19 20	30 18
Lu et al. (1992) [39]	Glioma in comparison to white matter	0.005–0.5	24 ± 0.5	30	30
Present study	Meningioma tissue in comparison to white matter tissue	0.5–18	25 ± 0.5	76.7	157.6
Present study	Meningioma tissue in comparison to gray matter tissue	0.5–18	25 ± 0.5	11.6	16.7

* The authors note that the recorded increase of 10.6% in the value of the imaginary part of permittivity of tumor tissues was not statistically significant due to a higher standard deviation of measurements. ** The comparison to normal fibroconnective/glandular tissue. *** The authors note that there was no statistical difference in the imaginary part of permittivity in the upper part of the measured frequency spectrum (closer to 10 GHz).

**Table 3:** Cole–Cole models parameters for the measured white matter and gray matter permittivity.

Tissue Type	Average Error		Maximum Error	
	$\varepsilon_{r}^{\prime }$	$\varepsilon_{r}^{\prime \prime }$	$\varepsilon_{r}^{\prime }$	$\varepsilon_{r}^{\prime \prime }$
White matter	1.4%	4.1%	3.3%	9.5%
Gray matter	1.5%	3.5%	6.6%	8.4%
Meningioma	0.5%	1.3%	6.4%	3.0%

## Data Availability

The measured permittivity data sets are available as Supplementary Materials accompanying this paper.

## Supplementary Materials

The following supporting information can be downloaded at: https://www.mdpi.com/article/10.3390/cancers15164153/s1. The measured permittivity values presented in Figure 7, Figure 8 and Figure 9 are made available as downloadable Supplementary Materials accompanying this paper. The raw data and the data after smoothing are given for each tissue. The measured data is accompanied by the permittivity calculated by (4), using the derived Cole–Cole models for each tissue, with the parameters from Table 1.
